# Current News

**Published:** 1892-04

**Authors:** 


### Current News.

### DENTAL SOCIETY OF THE STATE OF NEW YORK.

### PRELIMINARY NOTICE.

The Dental Society of the State of New York will hold its twenty-fourth annual meeting at Albany, Wednesday and Thursday, May 11 and 12, 1892.

Papers will be read by the following distinguished members of our profession: Edwin T. Darby, D.D.S., Philadelphia, Pa.; Eugene S. Talbot, D.D.S., Chicago, Ill.; C. F. W. Bödecker, D.D.S. New York City, N. Y.; Albert Carter Westlake, D.D.S., Elizabeth, N. J.; and discussed by prominent dentists from all parts of the United States. In order that the discussions may be interesting, all those who have been invited to open discussions will be allowed fifteen minutes, others ten.

Nothing will be left undone to promote the interests of practical and scientific dentistry.

NO CLINICS. NO EXHIBITS.

Please set aside the above time and do your share to advance the status of dentistry in the Empire State.

W. W. Walker, *President.*

C. S. Butler, *Secretary,*

67 West Unity Street,

### CENTRAL DENTAL ASSOCIATION OF NORTHERN NEW JERSEY.

At the meeting of the Central Dental Association of Northern New Jersey, held February 15, 1892, the following officers were elected:

President, Dr. Harvey Iredell, New Brunswick; Vice-President, Dr. R. M. Sanger, East Orange; Secretary, Dr. W. L. Fish, Newark; Treasurer, Dr. C. A. Meeker, Newark.

*Executive Committee.*—Dr. S. C. G. Watkins, Chairman, Montclair; Dr. George E. Adams, South Orange; Dr. F. C. Barlow, Jersey City; Dr. B. F. Luckey, of Patterson; Dr. Oscar Adelberg, Elizabeth.

Respectfully,

S. S. Hawley,
*Secretary.*

---

### HARVARD ODONTOLOGICAL SOCIETY.

The Fourteenth Annual Meeting and banquet of the Harvard Odontological Society, held at Young's Hotel, Boston, February 27, was one of unusual interest, owing in great measure to the number of distinguished gentlemen present as guests, and to the encouraging report of the chairman of the committee on the Harvard Dental School upon the matter of raising one hundred thousand dollars, the amount of money desired for the erection of a new hospital and school-building. Among the guests of the evening were Hon. Oliver Ames, Ex-Governor of Massachusetts; Henry P. Bowditch, M.D., Dean of the Medical School; Professor George A. Littlefield, A.B., Principal State Normal School, Rhode Island; Rev. David B. Jutten, D.D., of Boston; Thomas H. Chandler, D.M.D., Dean of the Dental School; Thomas Fillebrown, D.M.D., Professor of Operative Dentistry; Messrs. Albert A. Gleason, Fred. A. Stanley, of New Bedford; Babson S. Ladd, Esq., Dr. Nathaniel Greene, Dr. F. S. Faxon, Dr. V. C. Pond, Dr. S. A. Hopkins, and Dr. William Y. Allen, of Boston. Among those who were expected to be present, and from whom letters of regret were read, were Charles W. Eliot, LL.D., President of Harvard University; Clarence W. Barrow, Manager Boston News Bureau; Nathaniel S. Shaler, S.D., Professor of Geology, Harvard University; Francis A. Harris, A.B., M.D., State Medical Examiner, Massachusetts.

Previous to the banquet the annual reports were read and accepted, all of which showed the society to be in a most active and flourishing condition.

The following named gentleman were elected to serve the Society for the ensuing year:

President, Jere. E. Stanton, M.D., D.M,D., Boston; Recording Secretary, Waldo E. Boardman, D.M.D., Boston; Corresponding Secretary, Charles H. Taft, A.B., D.M.D., Cambridge; Treasurer, Dwight M. Clapp, D.M.D., Boston; Editor, Henry L. Upham, D.M.D., Boston.

*Executive Committee.*—Waldo E. Boardman, D.M.D., Boston; W. E. Page, D.M.D., Boston; Henry M. Clifford, D.M.D., Boston.

Dr. Eugene H. Smith was chosen to deliver the address at the next annual meeting.

When cigars had been lighted, and both members and guests had settled back to enjoy the intellectual feast to follow, President Stanton rapped to order, and in a few well-chosen words extended to the guests, on behalf of the Society, a hearty welcome and cordial appreciation of their interest in the Society as made manifest by their presence.

He then introduced Dr. Henry W. Gillette, of Newport, R. I., as the orator of the evening, whose address was a most thoughtful and scholarly effort, and which was listened to with marked attention by all present.

Dr. Dwight M. Clapp was then called upon for a brief report of the joint committee on the School and of the Faculty, as to the plans proposed for the raising of the necessary funds for the new building. He stated that a circular letter had been sent to every graduate of the Dental School, outlining the present and future needs of the school, which its growth, both as to number of students and patients, made necessary the taking of some immediate action.

The building now occupied by the school is the one formerly used by the Medical School, and in many respects is entirely unsuited for its present purposes; and instead of laying out money on the present building, which would be an unwise financial investment, inasmuch as it would still remain the property of the Medical School, it has been thought best to erect an entirely new building, providing the necessary funds can be raised.

Before calling upon the general public to assist in the undertaking, it is first proposed to ask the alumni for five thousand dollars ($5000), each alumnus to contribute such sum as he can afford; the same to be payable within sixty days to the treasurer of the university as soon as the sum of fifty thousand dollars ($50,000) shall have been raised or subscribed. A copy of the architect's design of the proposed new building had also been enclosed in the letter to each graduate.

The interest of the alumni in the matter was manifested in the subscriptions which have already been received within the last ten days, amounting to thirteen hundred dollars.

Through the generosity of an anonymous friend, who has within the past two years given different sums amounting in all to thirteen thousand dollars, the school now has, with its other funds, a balance of nearly twenty-one thousand dollars ($21,000) in its treasury.

The speeches which followed Dr. Clapp's report were replete with wit and wisdom, and the meeting at a late hour adjourned with that feeling of encouragement and enthusiasm which good-fellowship and loyalty to Harvard, and all that concerns her welfare, never fails to inspire in the hearts of all her sons, whatever her need, whatever the occasion.

CHARLES H. TAFT,
*Corresponding Secretary.*

---

### COLLEGE COMMENCEMENTS.

### PENNSYLVANIA COLLEGE OF DENTAL SURGERY.

| | |
|---|---|
| Graduates | 103 |
| Matriculates | 207 |

### KANSAS CITY DENTAL COLLEGE.

| | |
|---|---|
| Graduates | 50 |

### OHIO COLLEGE OF DENTAL SURGERY.

| | |
|---|---|
| Graduates | 89 |
| Matriculates | 143 |

### UNIVERSITY OF MARYLAND. DENTAL DEPARTMENT.

| | |
|---|---|
| Graduates | 74 |

### PHILADELPHIA DENTAL COLLEGE.

| | |
|---|---|
| Graduates | 142 |
| Matriculates | 259 |

---

### DENTAL PATENTS.

[WE have made arrangements to have a list of the patents in dental appliances forwarded to us as issued. This will appear regularly hereafter.—ED.]

468,653.—Dental Plugger. George W. Geitz, New York, N. Y. Filed May 21, 1891. Serial No. 393,514.

468,746.—Dental Pliers. Woodbury S. How, Philadelphia, assignor to the S. S. White Dental Manufacturing Company, same place. Filed December 3, 1891. Serial No. 413,898.

468,761.—Artificial Denture. Emory A. Bryant, Aspen, Col., assignor of one-half to Edward P. Rose, same place. Filed December 12, 1890. Serial No. 374,517.

468, 922.—Artificial Teeth. Rufus G. Stanbrough, New York, N. Y., assignor to Robert Seaman, same place. Filed December 9, 1891. Serial No. 414,513.

468,923.—Dental Tool. Rufus G. Stanbrough, New York, N. Y., assignor to Robert Seaman, same place. Filed December 9, 1891. Serial No. 414,514.

469,884.—Dental Heater. Woodbury S. How, Philadelphia, Pa., assignor to the S. S. White Dental Manufacturing Company, same place. Filed January 19, 1892. Serial No. 418,546.

---

### MEETING OF ALUMNI OF THE DEPARTMENT OF DENTISTRY, UNIVERSITY OF PENNSYLVANIA.

The Twelfth Annual Meeting of the Society of the Alumni of the Department of Dentistry, University of Pennsylvania, will be held at the Stratford Hotel, on Thursday, May 5, at 2 P.M. The annual supper to take place in the evening of the same day, at the same place.

Members who wish a place at the supper will please notify the secretary as early as possible.

W. L. Winner,
*Secretary.*

